# Cost-effectiveness of abatacept, rituximab, and TNFi treatment after previous failure with TNFi treatment in rheumatoid arthritis: a pragmatic multi-centre randomised trial

**DOI:** 10.1186/s13075-015-0630-5

**Published:** 2015-05-22

**Authors:** Sofie HM Manders, Wietske Kievit, Eddy Adang, Herman L Brus, Hein J Bernelot Moens, Andre Hartkamp, Lidy Hendriks, Elisabeth Brouwer, Henk Visser, Harald E Vonkeman, Jos Hendrikx, Tim L Jansen, Rene Westhovens, Mart AFJ van de Laar, Piet LCM van Riel

**Affiliations:** Department of IQ Healthcare, Radboud Institute for Health Sciences, Radboud University Medical Centre, Postbus 9101, Geert Grooteplein Noord 21 (Route 114), 6500 HB Nijmegen, Netherlands; Department of Health Evidence, Radboud University Medical Centre, Nijmegen, Netherlands; Department of Rheumatology, TweeSteden Hospital, Dr. Deelenlaan 5, Tilburg, 5042 AD Netherlands; Department of Rheumatology, Ziekenhuisgroep Twente, Zilvermeeuw 1, Almelo, 7609 PP Netherlands; Department of Rheumatology, Jeroen Bosch Hospital, Henri Dunantstraat 1, ’s-Hertogenbosch, 5223 GZ Netherlands; Department of Rheumatology, Medical Centre Leeuwarden, Henri Dunantweg 2, Leeuwarden, 8934 AD Netherlands; Department of Rheumatology, University Medical Centre Groningen, Hanzeplein 1, Groningen, 9700 RB Netherlands; Department of Rheumatology, Rijnstate Hospital, Postbus 9555, Arnhem, 6800 TA Netherlands; Department of Rheumatology and Clinical Immunology, Medisch Spectrum Twente, Haaksbergerstraat 55, Enschede, 7513 ER Netherlands; Department of Rheumatic Diseases, Radboud University Medical Centre, Nijmegen, Netherlands; Skeletal Biology and Engineering Research Centre, Department of Development and Regeneration, KU Leuven, Oude Markt 13, Leuven, 3000 Belgium; Department of Rheumatology, University Hospitals Leuven, Oude Markt 13, Leuven, 3000 Belgium

## Abstract

**Introduction:**

For patients with rheumatoid arthritis (RA) whose treatment with a tumour necrosis factor inhibitor (TNFi) is failing, several biological treatment options are available. Often, another TNFi or a biological with another mode of action is prescribed. The objective of this study was to compare the effectiveness and cost-effectiveness of three biologic treatments with different modes of action in patients with RA whose TNFi therapy is failing.

**Methods:**

We conducted a pragmatic, 1-year randomised trial in a multicentre setting. Patients with active RA despite previous TNFi treatment were randomised to receive abatacept, rituximab or a different TNFi. The primary outcome (Disease Activity Score in 28 joints) and the secondary outcomes (Health Assessment Questionnaire Disability Index and 36-item Short Form Health Survey scores) were analysed using linear mixed models. Cost-effectiveness was analysed on the basis of incremental net monetary benefit, which was based on quality-adjusted life-years (calculated using EQ-5D scores), and all medication expenditures consumed in 1 year. All analyses were also corrected for possible confounders.

**Results:**

Of 144 randomised patients, 5 were excluded and 139 started taking abatacept (43 patients), rituximab (46 patients) or a different TNFi (50 patients). There were no significant differences between the three groups with respect to multiple measures of RA outcomes. However, our analysis revealed that rituximab therapy is significantly more cost-effective than both abatacept and TNFi over a willingness-to-pay range of 0 to 80,000 euros.

**Conclusions:**

All three treatment options were similarly effective; however, when costs were factored into the treatment decision, rituximab was the best option available to patients whose first TNFi treatment failed. However, generalization of these costs to other countries should be undertaken carefully.

**Trial registration:**

Netherlands Trial Register number NTR1605. Registered 24 December 2008.

**Electronic supplementary material:**

The online version of this article (doi:10.1186/s13075-015-0630-5) contains supplementary material, which is available to authorized users.

## Introduction

In many countries, a tumour necrosis factor inhibitor (TNFi) such as adalimumab, certolizumab, etanercept, golimumab or infliximab is indicated for treating patients with rheumatoid arthritis (RA) who have moderate to high disease activity and whose treatment with methotrexate and at least one other conventional synthetic disease-modifying antirheumatic drug (csDMARD) has failed. However, according to the European League Against Rheumatism (EULAR), 30% to 50% of all patients treated initially with a first TNFi do not respond to treatment (that is, treatment failure); moreover, 20% to 45% of all patients discontinue treatment within 1 year [1-3]. Until recently, the best treatment option after previous TNFi treatment failure was to begin therapy with a different TNFi, a strategy that has proven effective regardless of the reason for the change in treatment [4-10]. However, after the failure of one TNFi, biologic disease-modifying antirheumatic drugs with a different mode of action can also be considered. Examples of these include abatacept, rituximab and tocilizumab. These biologic agents have high clinical effectiveness compared with placebo and csDMARDs. In addition, they have a good safety profile, [11-13] and are approved for RA treatment in the Netherlands and Belgium. In a recent study, researchers indirectly compared randomised controlled trials and found no difference in effectiveness between abatacept, rituximab and a second TNFi therapy in patients whose treatment with a first TNFi failed [14,15]. However, a difference in effectiveness cannot be excluded with certainty, as no direct comparison was performed, and all three treatment options differ with respect to their target and mechanism of action. Moreover, differences in the mode and frequency of delivery can result in a considerable variation in treatment costs. For example, abatacept and TNF inhibitors have fixed frequencies of administration, whereas rituximab can be given every 6 months or as dictated by disease activity. The objective of this study was to compare the effectiveness and cost-effectiveness of three treatment regimens (abatacept, rituximab or a different TNFi) after previous TNFi treatment failure in patients with RA.

## Methods

### Study design

A pragmatic multicentre randomised trial was performed to compare the effectiveness and cost-effectiveness of treating patients with abatacept, rituximab or a TNFi. Tocilizumab was not included, because it was not licensed at the start of the study. Using a web-based programme, patients with RA whose previous treatment with a TNFi failed were randomly assigned to receive rituximab, abatacept or a different TNFi (at a randomisation ratio of 1:1:1). Treatment failure was defined as either the physician or patient terminating the initial TNFi treatment. The reasons to terminate treatment included ineffectiveness or the onset of adverse events. In the randomisation protocol, each hospital was considered as a stratum, because patients in academic, teaching and general hospitals could be different and should be equally divided. Thereafter, treatment was not set to a specific protocol and was therefore provided at the discretion of the treating physician. Consistent with a pragmatic trial, care providers, participants and assessors were not blinded with respect to the treatment given. This study was approved by the Arnhem-Nijmegen regional ethics committee.

### Participants

Patients who met the following criteria were included in the study: previous treatment failure with their first TNFi, moderate to high disease activity (Disease Activity Score in 28 joints (DAS28) >3.2) and no previous treatment with abatacept or rituximab. Patients were excluded if they had a contraindication for treatment (for example, pregnancy, the presence of a serious infection) based on the rheumatologist’s judgment of if they had a strong preference or dislike for one of the treatment agents or did not want to be randomized. Patients were included between 2009 and 2012. Each patient signed an informed consent form for participation in this study.

### Interventions

The intervention was a treatment protocol using abatacept, rituximab or a different TNFi than the previous TNFi treatment. At the time of this study, five TNF inhibitors were available. The choice of TNFi was left to the discretion of the patient in the TNFi group and the patient’s treating physician. In general, the patients began their treatment with the Dutch or Belgian registered dose as follows: adalimumab was administered at 40 mg every 2 weeks; etanercept was administered at 50 mg per week or 25 mg twice per week; infliximab was administered at 3 mg/kg every 8 weeks after a loading dose given at weeks 0, 2 and 6; golimumab was administered at 50 mg every 4 weeks; and 400 mg of certolizumab was administered in weeks 0, 2 and 4, followed by a 200-mg dose given every 2 weeks. The dose of abatacept was based on the patient’s body weight as follows: patients who weighed <60 kg received 500 mg, patients weighing 60 to 100 kg received 750 mg and patients who weighed >100 kg received 1,000 mg. The doses were delivered by infusion over 1 hour every 4 weeks. Rituximab was administered by infusion (1,000 mg) at weeks 0 and 2. A second course could be administered after 6 months in patients who responded to the first course. The timing of the retreatment course depended on the increase in disease symptoms and activity and was at the discretion of the physician and patient. All treatment options could be administered in combination with a csDMARD or corticosteroid.

### Outcomes

The primary outcome for effectiveness was the DAS28 [16] over time. The secondary outcome measures were functional ability measured using the Health Assessment Questionnaire Disability Index (HAQ-DI) [17] and generic descriptive quality of life (36-item Short Form Health Survey (SF-36)) [18]. All effectiveness outcomes were measured at 0, 3, 6, 9 and 12 months. The outcomes for the cost-effectiveness analysis were medication costs and quality-adjusted life-years (QALYs) over a 12-month period. QALYs were based on utilities (a numeric value ranging from 0 to 1.0, reflecting health status: 0 indicates death, and 1.0 indicates the best health imaginable) calculated using the trapezium rule. The trapezium rule is a way of estimating the area under a curve. We know that the area under a curve is given by integration, so the trapezium rule gives a method of estimating integrals. The trapezium rule works by splitting the area under a curve into a number of trapeziums whose area we know [19]. The utilities were calculated using the EQ-5D score [20]. The EQ-5D is a generic measure of health status that provides a simple descriptive profile and a single index value that can be used in the clinical and economic evaluation of health care and in population health surveys. This questionnaire consists of five questions attending to five dimensions: mobility, self-care, usual activities, pain/discomfort and anxiety/depression. Each dimension has three levels: no problems, some problems and extreme problems [20,21]. Furthermore, all medication-related information was recorded, including the type of medication, start date, dose, stop date, change in medication and/or dose, and the reason for the change. Medication prices were obtained from the Dutch National Information Centre [22] in October 2013 and from the hospital pharmacist at Radboud University Medical Centre, Nijmegen, the Netherlands. All prices were adjusted to 2013, and additional costs were calculated for the infusion costs for the intramural treatments with full-cost pricing (see Additional file 1).

### Sample size

The three treatment options (abatacept, rituximab and TNFi) were considered equivalent if the two-sided 95% confidence interval (CI) showed that the treatments were at least not more than 0.4 DAS28 values better or worse than the comparator, using analysis of covariance on the mean DAS28 of 6, 9 and 12 months. The equivalence margin of 0.4 is one-third of a population standard deviation (SD = 1.2) and two-thirds of a minimal clinically important difference (0.6) for an individual patient [23]. Efficiency can be gained by analysing repeated measures; therefore, the SD can be adjusted using the correlation between the repeated measures [24]. The correlation was 0.70 between repeated DAS28 measures based on results from an inception cohort of patients with newly diagnosed RA, and the SD was 1.2 [24,25], which results in a SD of 0.67. This SD is used in a standard formula for noninferiority power calculations for continuous outcomes [26], leaving 44 evaluable patients per group required for 80% power to show noninferiority within the margin of 0.4 (see [27]).

### Statistical analyses

The results were analysed in accordance with the intention-to-treat principle, meaning that all patients were analysed in the medication group in which they initially started, regardless of whether they received and/or adhered to that treatment for the full 12 months. We excluded patients who were initially assigned to a treatment group but chose to continue taking their first TNFi treatment, patients who chose not to participate in the study and patients who developed a contraindication (and therefore no longer fulfilled the selection criteria).

The primary clinical outcome (mean DAS28) was analysed over time using linear mixed models. If a DAS28 score was missing because the erythrocyte sedimentation rate (ESR) was missing, the ESR was imputed based on other ESR values, the swollen joint count score, the tender joint count score, the Visual Analogue Scale score of the patient’s general health, age, and sex [28]. To handle missing baseline measurements (and therefore increase power), the missing indicator method was used in linear mixed models analyses [29]. Because there was coincidental unbalanced allocation in some observed variables, both corrected and uncorrected analyses were performed. In the corrected analyses, variables were added to the linear mixed model if they had a *P*-value <0.2 in the univariate analyses (one-way analysis of variance, Kruskal-Wallis test or χ^2^ test) and if they changed the β-coefficient of the variable ‘medication’ (abatacept, rituximab or TNFi) by >10% (a standard rule of thumb for this type of analysis). The analyses were repeated for the HAQ-DI, the SF-36 component scales and the EQ-5D scores. Moreover, the percentages of patients in remission (DAS28 <2.6) and low disease activity (DAS28 <3.2 and >2.6) and the percentages of patients with a good or moderate EULAR response were analysed at 6 and 12 months [30].

‘Drug survival’ (that is, time to drug discontinuation) was analysed with Kaplan-Meier and Cox proportional hazards models to correct for possible confounders. Drug survival was analysed only between TNFi and abatacept, because rituximab follow-up infusions were given on demand and a stop date was difficult to define.

A cost-effectiveness analysis was performed based on a time frame of 1 year. Medication costs are believed to be the principal incremental cost drivers in RA care for patients who use biologic treatments. Therefore, the costs used in the economic evaluation were based on the costs of the medications and the costs associated with intramural infusion of infliximab, rituximab or abatacept (see Additional file 1). The net monetary benefit (NMB) statistic was used because this is a regression-based approach to analysis of cost-effectiveness and can correct for confounders. NMB can be calculated as follows: NMB = (WTP × Effects) − Costs, where WTP is willingness to pay (in euros). Incremental NMB (INMB) is the difference between one NMB and another NMB. We used the following five threshold values for WTP for each QALY gained: 0, 20,000, 40,000, 60,000 and 80,000 euros. According to the decision rule, the option with the highest NMB is the most cost-effective, given that specific WTP. Confounders were taken into account as described above. The results were based on a general linear model that was bootstrapped 1,000 times to correct for uncertainty.

## Results

**Figure 1:**
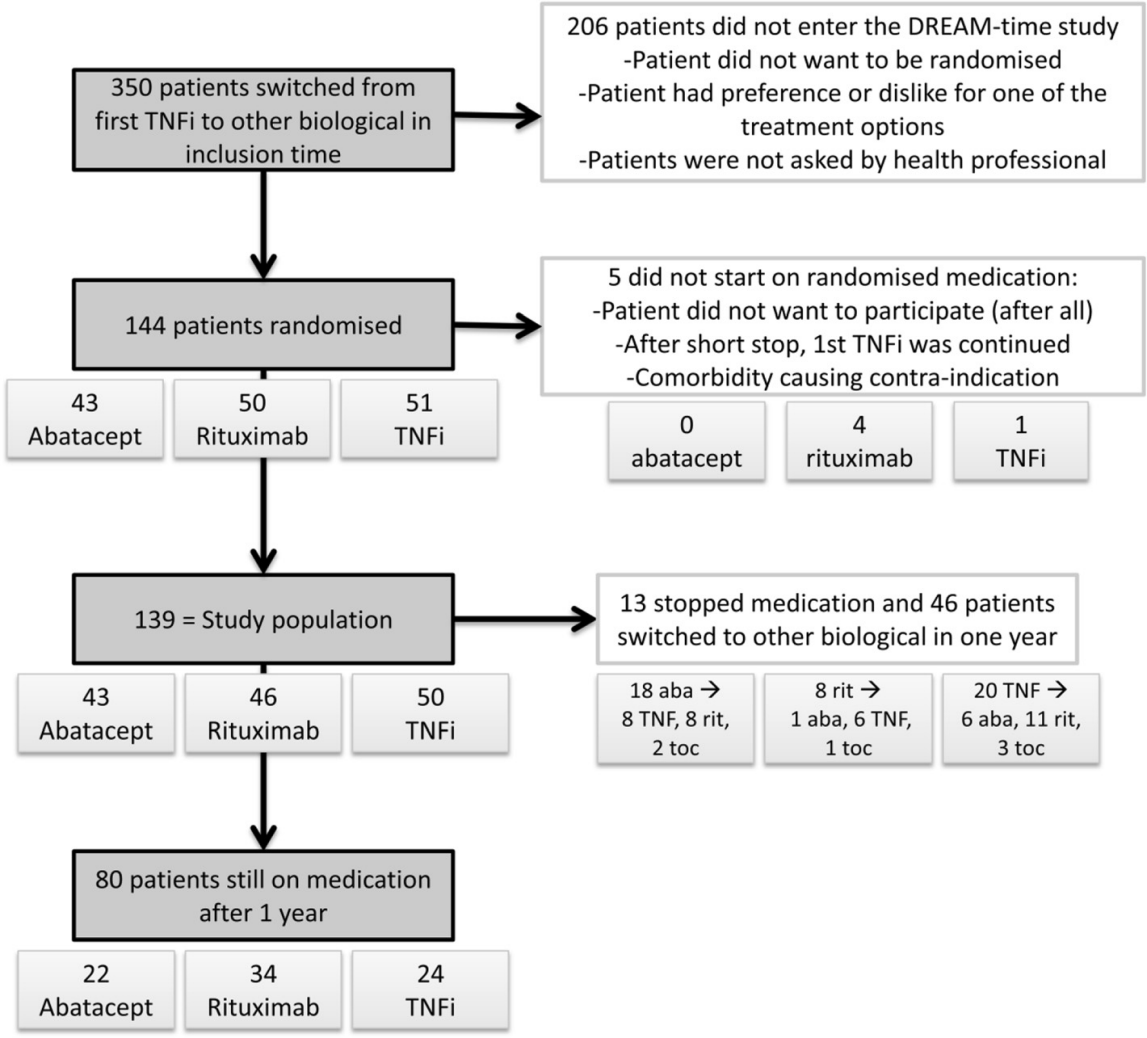
Inclusion and exclusion of patients in the study. aba, abatacept; rit, rituximab; TNF, Tumour necrosis factor; TNFi, Tumour necrosis factor inhibitor; toc, tocilizumab.

In our study, we randomly assigned 144 patients to one of three treatment arms—abatacept, rituximab or TNFi—and 206 patients were switched to another biologic treatment in the same period but did not participate in the study. Figure 1 provides an overview of patient selection, as well as the exclusion criteria that were applied throughout the study. Five patients did not begin taking their randomly assigned treatment; the reasons are shown in Figure 1. Therefore, 139 patients were ultimately included in the analyses. The TNFi group (n = 50) included patients who took adalimumab (n = 21), etanercept (n = 19), infliximab (n = 5), golimumab (n = 3) or certolizumab (n = 2). Figure 1 also shows that 59 patients stopped taking the medication or switched from their assigned treatment within 1 year—35 due to ineffectiveness, 14 due to side effects and 5 for other reasons—with median (interquartile range) durations on therapy of 6 (3 to 8) months, 2 (1 to 7) months and 6 (3 to 8) months, respectively. The patient baseline characteristics and comedications are summarised in Table 1. Both rheumatoid factor (RF) and sex differed between the three groups (*P* <0.2) and were therefore seen as possible confounders.

**Table 1:** **Patient characteristics at baseline** ^**a**^

		**Abatacept (n = 43)**	**Rituximab (n = 46)**	**TNFi (n = 50)**	**Total (n = 139)**	***P*** **-value**
Mean age (SD), yr		56.16 (9.95)	57.09 (11.08)	55.81 (12.53)	56.34 (11.24)	0.852
Female sex, %		88.4 (n = 38)	63.0 (n = 29)	74.0 (n = 37)	74.8 (n = 104)	0.022
Median disease duration (IQR), yr		6.56 (2.56 to 11.96)	7.60 (3.22 to 16.25)	5.64 (1.79 to 12.00)	6.25 (2.43 to 14.30)	0.174
RF-positive, %		56.4 (n = 22)	80.0 (n = 36)	62.5 (n = 30)	66.7 (n = 88)	0.054
Mean DAS28 (SD)		4.74 (1.46)	4.87 (1.24)	4.92 (1.11)	4.84 (1.26)	0.805
Mean HAQ-DI (SD)		1.46 (0.64)	1.39 (0.71)	1.37 (0.65)	1.40 (0.66)	0.822
Median previous csDMARDs (IQR), n		2 (2 to 3)	3 (2 to 3)	2 (2 to 3)	2 (2 to 3)	0.192
Comedication, %						0.894
	csDMARD + Corticosteroid^b^	20.9 (n = 9)	28.3 (n = 11)	28.0 (n = 14)	25.9 (n = 36)	
	csDMARD^b^	41.9 (n = 18)	50.0 (n = 23)	38.0 (n = 19)	43.2 (n = 60)	
	Corticosteroid	11.6 (n = 5)	8.7 (n = 4)	10.0 (n = 5)	8.6 (n = 12)	
	None (biologic monotherapy)	25.6 (n = 11)	17.4 (n = 8)	24.0 (n = 12)	22.3 (n = 31)	

^a^csDMARD, Conventional synthetic disease-modifying antirheumatic drug; DAS28, Disease Activity Score in 28 joints; HAQ-DI, Health Assessment Questionnaire Disability Index; IQR, Interquartile range; RF, Rheumatoid factor; SD, Standard deviation. ^b^Of the patients who took the biologic in combination with a csDMARD, methotrexate was the comedication in 75.0% of patients in the abatacept group, in 91.4% in the rituximab group and in 86.0% in the TNFi group.

### Treatment effectiveness

**Figure 2:**
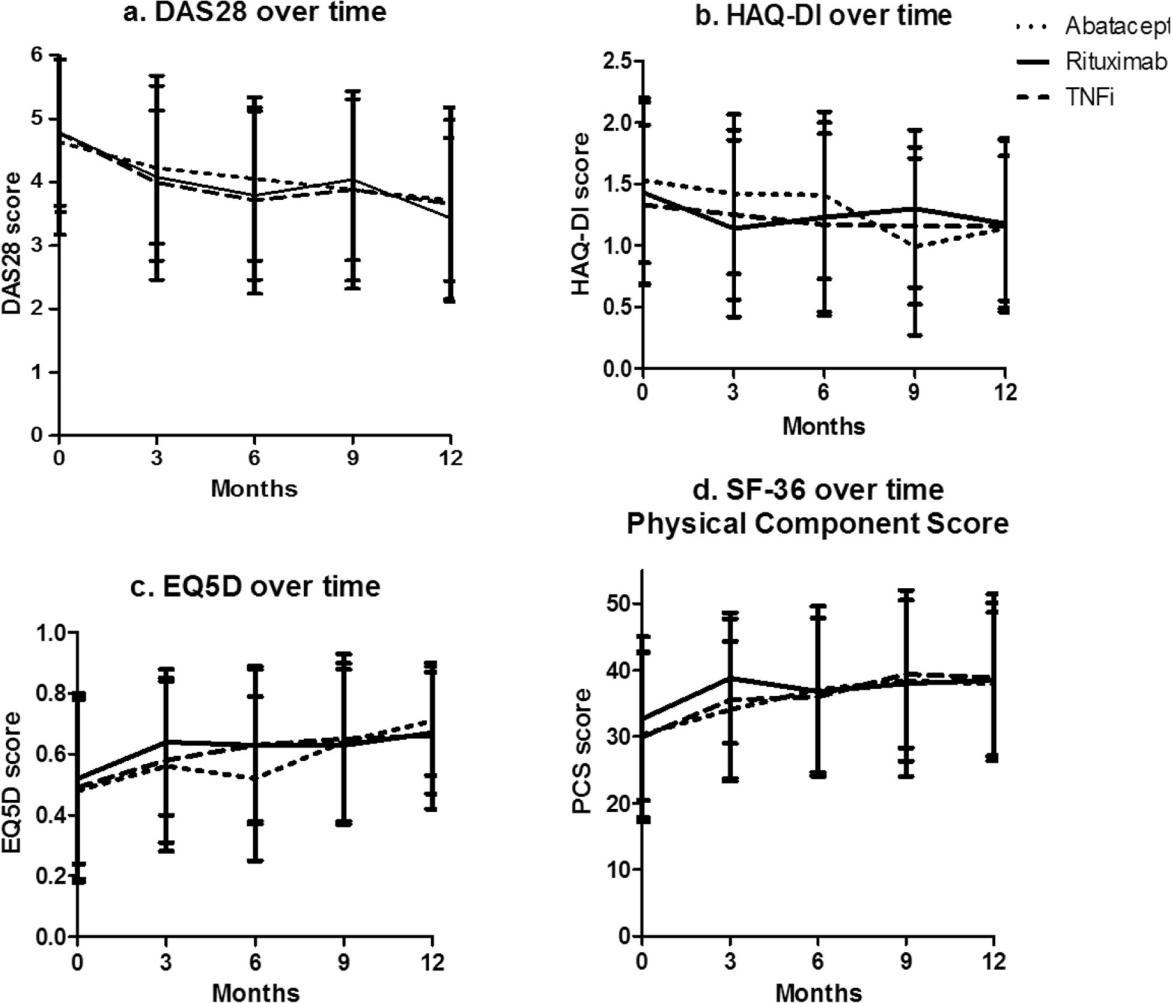
Effectiveness outcomes over time with standard deviations. **(a)** Disease Activity Score in 28 joints (DAS28). **(b)** Health Assessment Questionnaire Disability Index (HAQ-DI). **(c)** EQ-5D. **(d)** 36-item Short-Form Health Survey (SF-36). PCS, Physical Component Summary. Month 0 represents the start of the treatment.

**Figure 3:**
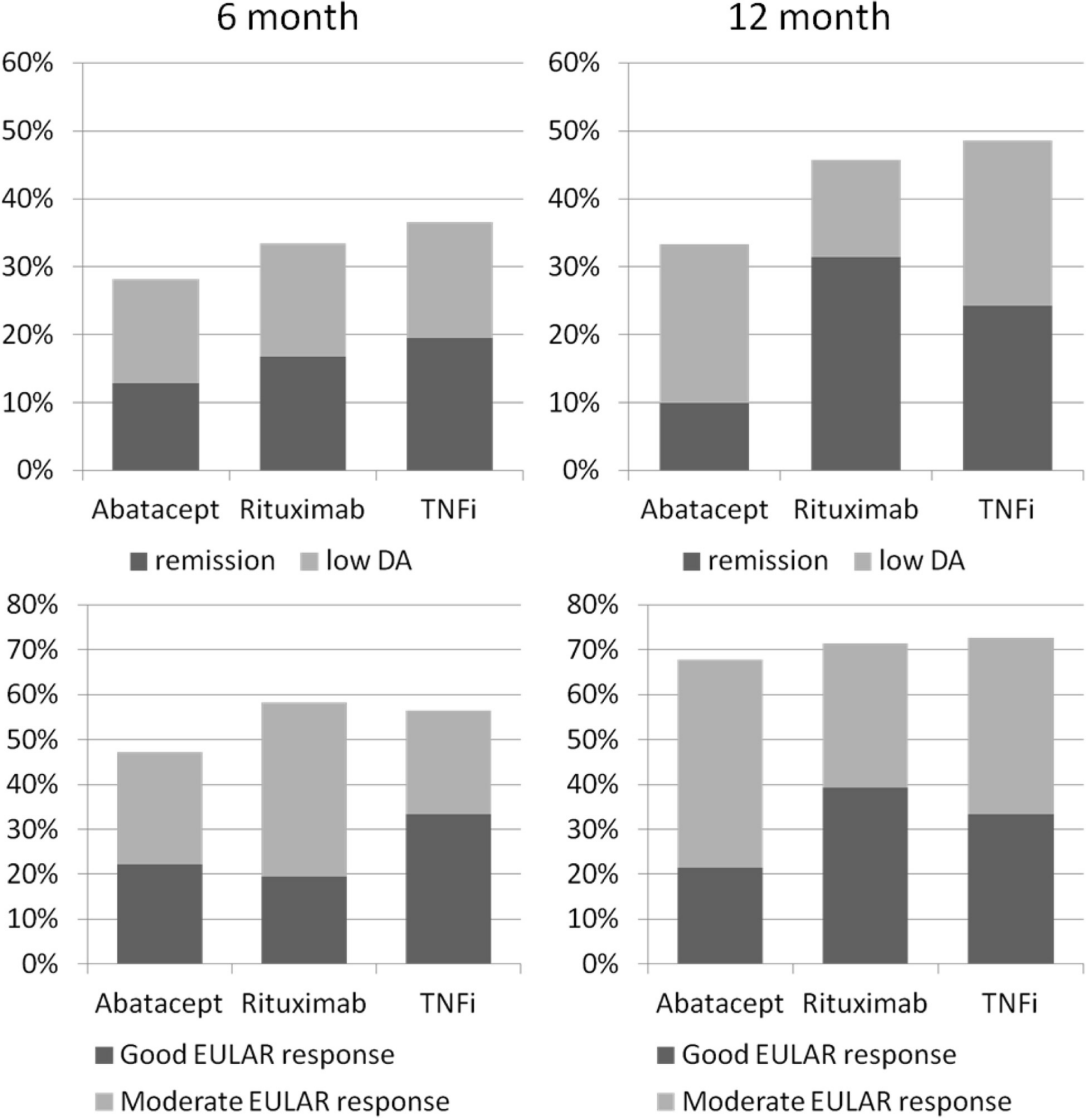
Percentages of patients in remission, with low disease activity and with good or moderate European League Against Rheumatism response criteria [30]. DA, Disease activity; EULAR, European League Against Rheumatism; TNFi, Tumour necrosis factor inhibitor.

The mean (SD) DAS28 scores at 12 months were 3.8 (1.2) for abatacept, 3.4 (1.2) for rituximab and 3.5 (1.5) for TNFi (Figure 2a). Figure 3 presents the percentage of patients in remission, low disease activity and EULAR good or moderate response at 6 and 12 months for the three groups. There were no significant differences between the three treatment groups with respect to DAS28, HAQ-DI, EQ-5D or SF-36 over time (analysed using linear mixed models that were either uncorrected or corrected for RF and sex) (see Figure 2).

### Drug survival

The 1-year drug survival between TNFi and abatacept was not significantly different for either the corrected analyses (expβ = 0.697, 95% CI = 0.369 to 1.318, *P* = 0.267) or the uncorrected analyses (expβ = 0.870, 95% CI = 0.489 to 1.546, *P* = 0.635).

### Safety

Fifty-one patients (36.7%) in the study reported at least one adverse event at 1 year (16 in the abatacept group, 15 in the rituximab group and 20 in the TNFi group) (see Table 2). One suspected unexpected serious adverse reaction (SUSAR) occurred within 1 year. This patient, who was in the abatacept group, became psychotic 4 months after the start of the study. However, in retrospect, this SUSAR did not appear to be related to the medication.

**Table 2:** **Adverse events reported at the 1-year follow-up examination**

	**Abatacept (n = 43)**		**Rituximab (n = 46)**		**TNFi (n = 50)**		**Total (n = 139)**	
	**Number of events**	**Number of patients**	**Number of events**	**Number of patients**	**Number of events**	**Number of patients**	**Number of events**	**Number of patients**
Cardiovascular event	1	1	2	2	2	2	5	5
Infection	9	6	5	4	11	7	25	18
Malignancy	0	0	3	3	0	0	3	3
Laboratory abnormalities^a^	1	1	0	0	2	2	3	3
Skin condition	3	3	3	3	6	5	12	11
Gastroenterological	2	2	3	3	0	0	5	5
Other^b^	5	4	7	5	7	4	19	13
Total	21	16	23	15	28	20	78	51

^a^Liver function test elevations and leukopenia. ^b^In this group, the adverse effects consisted primarily of influenza, fever, fatigue, headache and/or dizziness. One patient in the abatacept group developed psychosis 4 months after the start of the study.

### Cost-effectiveness

In our analysis of cost-effectiveness, the costs were dependent upon the medication used, the dose delivered, the method of delivery and the frequency of administration. In the abatacept group, 3% of the patients started with a 500-mg dose, 84% started with a 750-mg dose and 13% started with a 1,000-mg dose. All of these patients started treatment with a frequency of one dose every 4 weeks. All of the patients in the rituximab group started at the recommended dose of two doses of 1,000 mg. Eight patients stopped the rituximab treatment and switched to another biologic (see Figure 1), eight patients did not stop but also did not receive a second dose within 1 year, and thirty patients did receive a second dose. The mean interval between the first and second rituximab treatments was 9 months (11 patients received their second dose at 6 months). In the TNFi group, all of the patients except one started at the recommended dose and frequency; this patient took adalimumab weekly rather than once every 2 weeks.

**Figure 4:**
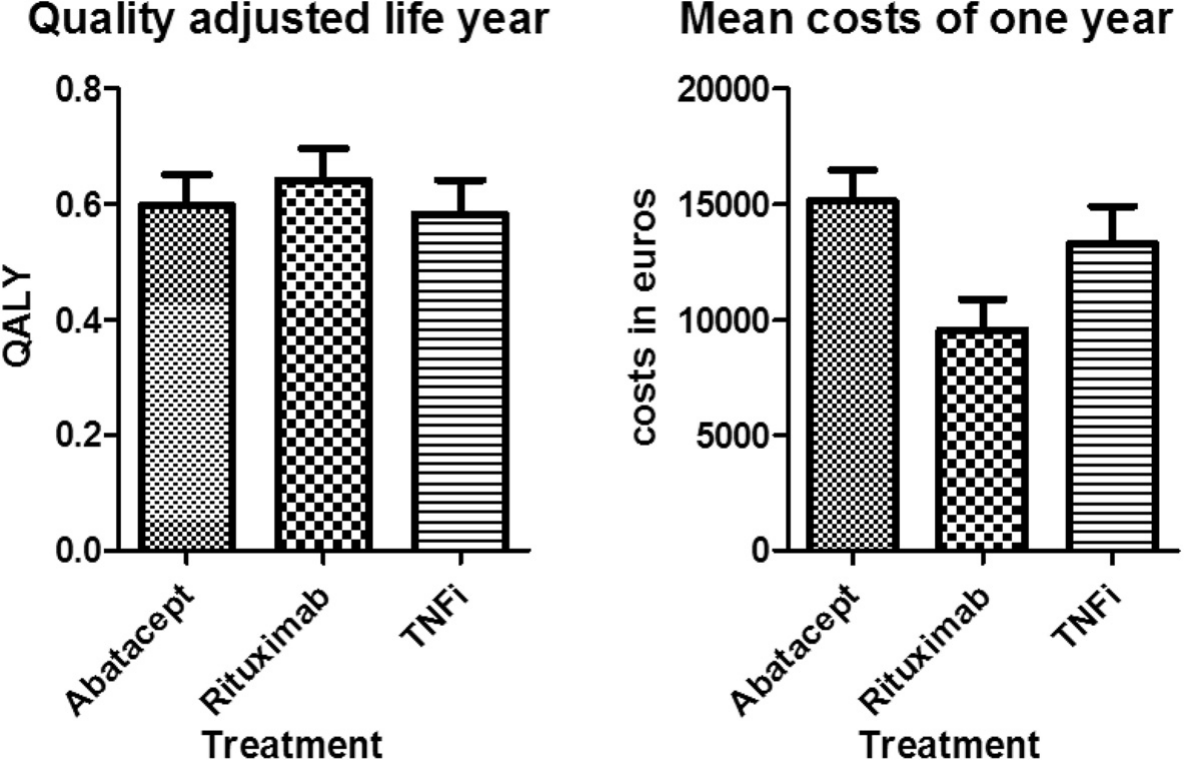
Mean quality-adjusted life-years and medication-related costs in a 1-year period. Error bars represent the upper bars of the 95% confidence intervals. QALY, Quality-adjusted life-year.

The 1-year mean QALYs and medication-related costs (in euros) are presented in Figure 4. The uncorrected difference in cost was significant between the abatacept and rituximab groups (mean difference = €5,586, 95% CI = €3,681 to €7,491, *P* <0.001) and between the TNFi and rituximab groups (mean difference = €3,758, 95% CI = €1,661 to €5,856, *P* = 0.001), but not between the TNFi and abatacept groups (mean difference = €1,828, 95% CI = −€294 to €3,950, *P* = 0.090).

**Figure 5:**
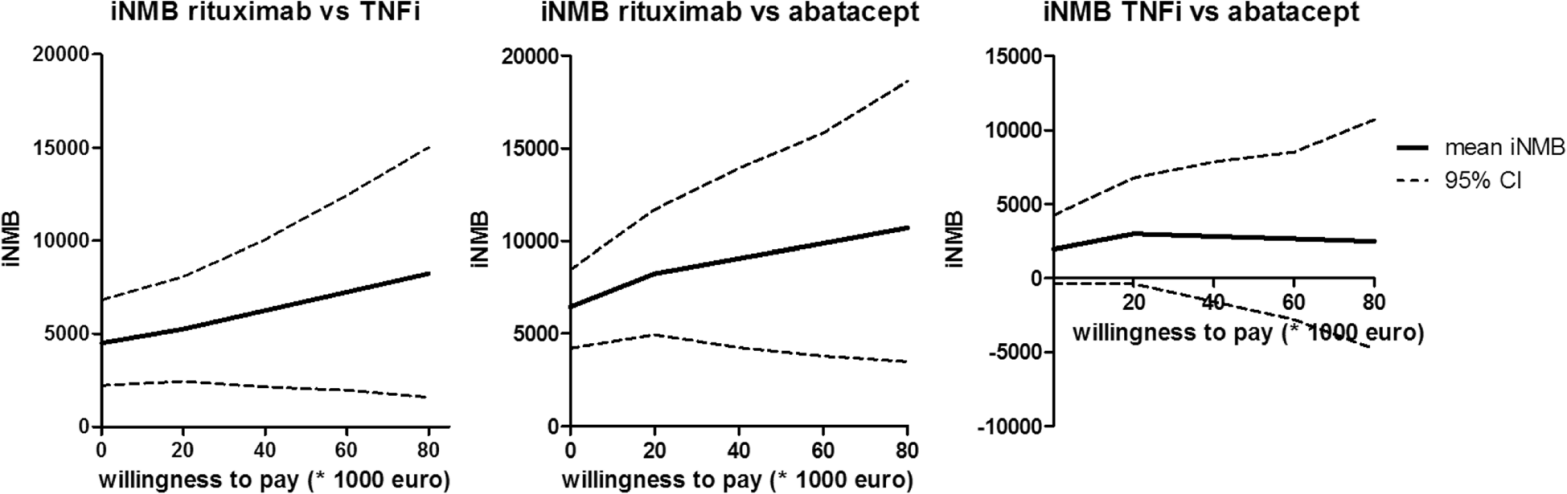
Mean incremental net monetary benefit (iNMB) with 95% confidence intervals (CIs). The location of the 95% CI lines below an INMB of zero indicates that the two treatment groups differed significantly with respect to cost-effectiveness.

A positive INMB can be interpreted as the added value (in euros) of following one treatment versus another. When uncorrected and corrected for sex and RF, over a WTP range of 0 to 80,000 euros per QALY, INMB was significantly different between the rituximab and abatacept groups (*P* <0.001) and between the rituximab and TNFi groups (*P* <0.05), as the INMB of zero did not fall within the 95% CI (that is, the zero data point on the *y*-axis was below the dashed lines) (see Figure 5). In contrast, the difference between abatacept and TNFi was not significant over the entire WTP range from 0 to 80,000 euros per QALY (that is, the lower 95% CI fell below 0 INMB over the entire WTP range). However, TNFi had a higher INMB than abatacept over the entire WTP range of 0 to 80,000 euros per QALY; thus, with a WTP of 0, 40,000 and 80,000 euros, the probability of TNFi being more cost-effective than abatacept was 97%, 75% and 50%, respectively. The analyses performed to calculate these percentages were uncorrected for confounders.

## Discussion

The objective of this study was to compare the effectiveness and cost-effectiveness of three standard treatments for RA—abatacept, rituximab and TNFi—in patients with RA whose previous TNFi treatments had failed. Our analysis revealed overall improvements over time, but no significant differences between the three treatment groups with respect to the DAS28, HAQ-DI or SF-36 outcome measures. However, we found significant differences in the costs and cost-effectiveness of the various treatments. Specifically, of the three treatments, rituximab was the most cost-effective. Moreover, treating patients with a second TNFi was more cost-effective than treating patients with abatacept.

This study provides important clinical insights, as it is the first study to directly compare three different treatment options for patients with RA whose first TNFi treatment failed. Our results are consistent with previous indirect and observational studies regarding the effectiveness of abatacept, rituximab and TNFi in patients with RA whose first TNFi treatment failed their [14,15,31]. However, Finckh *et al*. [32], in their observational study, concluded that changing to rituximab after the ineffectiveness of a previous TNFi is more effective than switching to an alternative TNFi. The results of the study by Finckh *et al*. can also be explained by the higher DAS28 at baseline in the rituximab group.

Our cost-effectiveness results are consistent with those of previous studies. For example, Malottki *et al*. in the United Kingdom and Hallinen *et al*. in Finland used QALYs to analyse cost-effectiveness and also concluded that rituximab was the most cost-effective treatment [33,34]. However, in a recent review of the cost-effectiveness of abatacept, Athanasakis *et al*. reported inconclusive results regarding the comparative cost-effectiveness of abatacept versus rituximab [35]. Their review also included studies that cannot be compared directly with our study; for example, some studies used an outcome variable other than QALYs. In our analysis, medication-related costs were dependent upon the price, dose, route of delivery and dosing frequency of the medication. These factors should be taken into account when attempting to compare our cost-effectiveness data with data from other countries.

A strength of our study lies in its pragmatic, randomised, multicentre design [36], a design that provides a suitable combination of internal and external validity [37]. After randomisation (which minimises the potential confounding by indication), treatments are provided as normal daily clinical practice, which is often different from strict protocols in controlled trials [3,38]. Thus, the external validity is maximised. Moreover, in this multicentre study, patients were recruited from a variety of hospitals (including academic, general and teaching hospitals) throughout the Netherlands and Belgium, with no strict inclusion or exclusion criteria. Therefore, the results of this study can be generalised to a broader population of patients with RA whose first TNFi treatment failed.

The generalisability of the cost-effectiveness is more difficult because every country has its own agreements about treatment prices. However, the main cost driver is the cost per milligram of (biologic) treatment. By using the data regarding doses, frequencies, means of delivery and prices of the medications provided in this article, costs can be calculated for other countries and compared with our results.

This study also has some limitations. First, we did not include tocilizumab therapy as a treatment arm, as this treatment option was not licensed at the start of the study. Tocilizumab has been reported to be effective for treating RA [13], and its effectiveness is similar to that of other biologic treatments [39] when given to patients whose first TNFi treatment has failed [14,40]. The dose—and thus the cost—of administering tocilizumab is dependent upon body weight. For example, for a patient weighing 70 kg, the mean annual cost is approximately 15,000 euros, which is similar to the cost of abatacept. Thus, tocilizumab may be a good alternative to the treatment options evaluated in this study. However, tocilizumab has not been compared directly with other biologic medications for treatment of RA in patients whose first TNFi treatment failed, thus warranting the need for future studies. A second limitation is the unequal distribution of RF among the three treatment groups, despite our randomisation approach. Studies have shown that rituximab yields better results in RF-positive patients (a difference of 0.3 on the DAS28 score relative to RF-negative patients) [41,42]. In our study cohort, there were fewer RF-positive patients than usually seen in daily clinical practice. The rituximab group contained a higher percentage of RF-positive patients than the other two groups; therefore, the effects of rituximab might have been overestimated. However, in daily clinical practice, rituximab is given primarily to RF-positive patients. To control for this, we corrected for this difference in RF positivity in our analyses and obtained the same results for the corrected and uncorrected analyses. It would be interesting to perform subgroup analyses for RF-positive patients and RF-negative patients, but the subgroups were too small to make valid conclusions. With respect to abatacept and TNFi treatment, the published literature has yielded no evidence regarding different treatment effectiveness in RF-positive versus RF-negative patients [42,43]. A third caveat with regard to our study is that 22% of the patients used the biologic treatment as monotherapy. This is different from what is suggested in the literature and provides a possible explanation for the moderate effects over time of all three groups. However, owing to the pragmatic trial design, this is a good representation of daily clinical practice.

It is difficult to perform a pragmatic trial. As we can also see in this study, it is difficult to motivate patients to participate in pragmatic studies. Patients do not have to participate to receive the treatments that are studied. If they do not want to be randomised, or if they have a preference or dislike for one specific treatment, they do not want to be included in the study. Fortunately, in this study, the sample size was reached because multiple hospitals included patients. With regard to patient characteristics, the patients who did not participate in the study but did change their TNFi treatment did not differ from included patients. Another caveat that must be considered regards our method of calculating medication-related costs, which included only the medication and infusion costs in our cost-effectiveness analysis. However, we found no difference between the groups with respect to treatment effectiveness, and there seem to have been no major differences in adverse events. If we assume that health care–related expenses are positively related to both disease activity and the development of adverse events, we can also assume that other health care–related costs would be similar between the three treatment groups. Although travel costs are somewhat higher for patients who require infusion treatments (that is, the patients in the rituximab and abatacept groups), only the patients in the abatacept group received a large number of infusions. Nevertheless, had we included travel expenses, the cost of treatment in the abatacept group would have been higher. After this study was concluded, abatacept became available as an injection therapy that can be administered at home, reducing the administration cost by an estimated 6%; therefore, treatment with abatacept can become less expensive while providing the same effectiveness [44]. The average costs of rituximab are lower partly because it was provided on demand and on average a second treatment was not provided at 6 months. Still, the effectiveness was the same as that of abatacept and TNFi. Because we study daily clinical practice, these kind of variations are detected. Perhaps, in the current care paradigm, abatacept and TNFi can also be provided less frequently or in lower doses as stated in protocols. This will then also reduce the health care costs.

In our study, all of the treatment options (including the various TNF inhibitors) had a good safety profile, and the short-term (1 year) occurrence of adverse events seems similar between the abatacept, rituximab and TNFi treatment groups, but the numbers were too small to perform valid statistical analyses. Aaltonen *et al*. [45] showed no significant difference between TNFi and rituximab treatment in patients with serious infections and malignancies. To make valid conclusions about the adverse events, another study that is powered on the adverse events should be performed. With respect to long-term safety, van Vollenhoven *et al*. [46] recently reported that long-term biologic treatment did not increase the risk of any type of adverse events during 9.5 years of follow-up observations. This supports our assertion that the treatments studied here are also likely to be equally safe in the long run. In addition, in a recent study, researchers reported that the dose of rituximab can be decreased to a single 1,000-mg dose or two 500-mg doses [47] rather than two 1,000-mg doses, as prescribed in our study. This decrease in dose can decrease the cost of rituximab by almost 50% if the time to the second dose is the same, which would greatly benefit rituximab’s cost-effectiveness and perhaps decrease the adverse events. With the upcoming biosimilars for rituximab and TNF inhibitors, lower costs related to those treatments are to be expected.

## Conclusions

Compared with intravenous abatacept and the various TNF inhibitors that were tested in this study, rituximab is the most cost-effective treatment option for patients whose first TNFi treatment has failed. This advantage is due primarily to the differences in drug costs; thus, because the effectiveness and safety are the same, the costs of the medication can drive decision making about a biologic treatment. Considering the clinical effectiveness and costs of pharmacologic treatments after failure of the first TNFi in patients with RA over a 12-month period, we found that rituximab was the most favourable treatment.

This study should be considered as an early step that needs to be confirmed by similar analyses with larger populations, including tocilizumab, and followed over longer periods of time so that the social and financial costs of different treatment regimens are also accounted for, including adverse events and inconvenience for patients.

## Additional file

Additional file 1:
**Medication costs.**

## Abbreviations

aba: Abatacept
CI: Confidence interval
csDMARD: Conventional synthetic disease-modifying antirheumatic drug
DAS28: Disease Activity Score in 28 joints
ESR: Erythrocyte sedimentation rate
EULAR: European League Against Rheumatism
HAQ-DI: Health Assessment Questionnaire Disability Index
INMB: Incremental net monetary benefit
IQR: Interquartile range
QALY: Quality-adjusted life-year
RA: Rheumatoid arthritis
RF: Rheumatoid factor
rit: Rituximab
SD: Standard deviation
SF-36: 36-item Short Form Health Survey
SUSAR: Suspected unexpected serious adverse reaction
TNFi: Tumour necrosis factor inhibitor
toc: Tocilizumab
WTP: Willingness to pay
