# Supplementary material for: Cost-effectiveness of abatacept, rituximab, and TNFi treatment after previous failure with TNFi treatment in rheumatoid arthritis: a pragmatic multi-centre randomised trial
Source: Arthritis Res Ther. 2015 May 22;17(1):134. doi: 10.1186/s13075-015-0630-5 (PMC4489004; doi:10.1186/s13075-015-0630-5)
Supplement: Additional file 1: — Medication costs. [file 13075_2015_630_MOESM1_ESM.docx]

**Additional file 1: Medication Costs**

|  | Concentration (mg/ml) | Volume (ml) or unit | Cost (euros) | Cost per mg (euros) | Source |
| --- | --- | --- | --- | --- | --- |
| Adalimumab | 50 | 0.8 | 556.67 | 13.9168 | [www.medicijnkosten.nl](http://www.medicijnkosten.nl/) |
| Infliximab | 100 | 1 | 631.37 | 6.3137 | [www.medicijnkosten.nl](http://www.medicijnkosten.nl/) |
| Etanercept | 50 | 0.5 | 134.87 | 5.3948 | [www.medicijnkosten.nl](http://www.medicijnkosten.nl/) |
| Golimumab | 100 | 0.5 | 1151.76 | 23.0352 | [www.medicijnkosten.nl](http://www.medicijnkosten.nl/) |
| Tocilizumab | 20 | 10 | 407.72 | 2.0386 | [www.medicijnkosten.nl](http://www.medicijnkosten.nl/) |
| Certolizumab pegol | 200 | 1 | 515.99 | 2.5800 | [www.medicijnkosten.nl](http://www.medicijnkosten.nl/) |
| Rituximab i.v. | 10 | 10 | 290 | 2.9000 | Pharmacist at Radboud UMC |
| Abatacept i.v. | 250 | 1 | 373.85 | 1.4954 | Pharmacist at Radboud UMC |
| Abatacept s.c. | 125 | 1 | 263.09 | 2.1047 | [www.medicijnkosten.nl](http://www.medicijnkosten.nl/) |
|  |  |  |  |  |  |
|  |  |  |  |  |  |
| MTX tablet | 2.5 | 1 | 0.13 | 0.0520 | [www.medicijnkosten.nl](http://www.medicijnkosten.nl/) |
| MTX injection | 25 | 1 | 25.19 | 1.0074 | [www.medicijnkosten.nl](http://www.medicijnkosten.nl/) |
| Leflunomide | 10 | 1 | 1.16 | 0.1160 | [www.medicijnkosten.nl](http://www.medicijnkosten.nl/) |
| Hydroxychloroquine | 200 | 1 | 0.13 | 0.0007 | [www.medicijnkosten.nl](http://www.medicijnkosten.nl/) |
| Methylprednisolone | 40 | 1 | 2.03 | 0.0508 | [www.medicijnkosten.nl](http://www.medicijnkosten.nl/) |
| Sulfasalazine | 500 | 1 | 0.08 | 0.0002 | [www.medicijnkosten.nl](http://www.medicijnkosten.nl/) |
| Azathioprine | 25 | 1 | 0.14 | 0.0056 | [www.medicijnkosten.nl](http://www.medicijnkosten.nl/) |
| Prednisone | 2.5 | 1 | 0.25 | 0.1000 | [www.medicijnkosten.nl](http://www.medicijnkosten.nl/) |
| Prednisolone | 5 | 1 | 0.04 | 0.0080 | [www.medicijnkosten.nl](http://www.medicijnkosten.nl/) |
| Triamcinolone hexacetonide | 10 | 1 | 1.52 | 0.1520 | [www.medicijnkosten.nl](http://www.medicijnkosten.nl/) |
| Aurothiomalate | 100 | 0.5 | 47.16 | 0.9432 | [www.medicijnkosten.nl](http://www.medicijnkosten.nl/) |

**Cost for receiving an infusion: 87.43 euros per treatment.** This cost includes staff (secretary, nurse, and rheumatologist), materials (NaCI, miniplasco, needle, transfusion system, filter IV star, three-way stopcock/tap, etc.), equipment (infosomat, automatic blood pressure measuring instrument, etc.), and overhead costs.
